# Assessment of interprofessional competence in undergraduate health professions education: protocol for a systematic review of self-report instruments

**DOI:** 10.1186/s13643-020-01394-7

**Published:** 2020-06-12

**Authors:** Renée Allvin, Carl Thompson, Samuel Edelbring

**Affiliations:** 1 Clinical Skills Center, Örebro University Hospital, S 701 85 Örebro, Sweden; 2 School of Health Sciences, Örebro University, S-702 81 Örebro, Sweden; 3 School of Healthcare, University of Leeds, Leeds, LS2 1TJ UK

**Keywords:** Evaluation, Instruments, Interprofessional learning, Questionnaires, Surveys, Systematic review protocol, Undergraduate students

## Abstract

**Background:**

Health practitioners from different professions, and with differing competencies, need to collaborate to provide quality care. Competencies in interprofessional working need developing in undergraduate educational preparation. This paper reports the protocol for a systematic review of self-report instruments to assess interprofessional learning in undergraduate health professionals’ education.

**Methods:**

We will search PubMed, Web of Science, CINAHL and ERIC from January 2010 onwards. A combination of search terms for interprofessional learning, health professions, psychometric properties, assessment of learning and assessment tools will be used. Two reviewers will independently screen all titles, abstracts and full-texts. Potential conflicts will be resolved through discussion. Quantitative and mixed-methods studies evaluating interprofessional learning in undergraduate health professions education (e.g. medicine, nursing, occupational and physical therapy, pharmacy and psychology) will be included. Methodological quality of each reported instrument, underpinning theoretical frameworks, and the effects of reported interventions will be assessed. The overall outcome will be the effectiveness of instruments used to assess interprofessional competence. Primary outcomes will be the psychometric properties (e.g. reliability, discriminant and internal validity) of instruments used. Secondary outcomes will include time from intervention to assessment, how items relate to specific performance/competencies (or general abstract constructs) and how scores are used (e.g. to grade students, to improve courses or research purposes). Quantitative summaries in tabular format and a narrative synthesis will allow recommendations to be made on the use of self-report instruments in practice.

**Discussion:**

Many studies use self-report questionnaires as tools for developing meaningful interprofessional education activities and assessing students’ interprofessional competence. This systematic review will evaluate both the benefits and limitations of reported instruments and help educators and researchers (i) choose the most appropriate existing self-report instruments to assess interprofessional competence and (ii) inform the design and conduct of interprofessional competency assessment using self-report instruments.

**Systematic review registration:**

Open Science Framework [https://osf.io/vrfjn].

## Background

Healthcare is increasingly complex, often involving delivering care and treatment to ageing populations, with multiple comorbid conditions [1]. Thus, health practitioners from different professions, with differing competencies, need to collaborate to provide quality care. These interprofessional competencies (IPCs) need to be prepared and developed in undergraduate health professional education. Whilst theoretically straightforward, this has proven difficult within health educational programs of preparation [2].

Interprofessional learning occurs when students from two or more professions learn about, from and with each other, to enable effective collaboration and improve health outcomes [3]. Interprofessional education (IPE) is implemented in different healthcare contexts, often focusing on, but not limited to, teamwork and communication in medicine and nursing practice [4]. Other reviews of research have identified barriers and facilitators to IPE [4], mechanisms underpinning outcomes of IPE [5], effective teaching methods [6], learner outcome levels [7] and assessment tools applicable to specific national contexts [8]. The psychometric properties of assessment tools measuring IPE have also been evaluated [9, 10]. Attempts have also been made to explain how, why and when IPE is successful [4, 11]. The effects of IPE on learning outcomes across all health professions are inconclusive [12]. There is a need for deeper knowledge of how principles of IPC can be expressed in learning activities and assessment practice [9].

IPC has several aspects, reflecting the complex interactions between professionals that can be involved. The Interprofessional Education Collaborative (IPEC) framework describes IPCs using four dimensions: values/ethics for interprofessional practice, roles/responsibilities, interprofessional communication and teams and teamwork [13]. Because of the multidimensional characteristics and heterogeneous relationships to clinical situations, these competencies present a challenge to systematic assessment. Arguably, many aspects of IPE are best assessed in real-life clinical situations. However, while direct observation of actual interprofessional behaviour is preferable, observation is hindered by limited opportunities for observing students and scarcity of trained observers [14]. Consequently, the majority of interprofessional developmental activities use self-report questionnaires to assess IPCs. Many such tools are also being used in research studies of IPE, with some systematically derived estimates as high as 70% [15]. The psychometric quality of IPE assessment instruments has been questioned, and there are reasons to believe that there is room for improvement on how outcomes should be interpreted [16]. Whilst it is hard to conceive that self-report instruments alone would ever provide valid and reliable measures of IPC, if used wisely, they can be a valuable part of IPE assessment strategies. Thus, there is a need to identify variations in the characteristics of assessment tools, the ways they are used and their effects on the educational outcomes they are intended to foster. This systematic review aims to contribute to knowledge and assessment practices surrounding IPL in undergraduate health professional education.

To achieve this aim, we have four objectives:

1. Determine the quality of self-report instruments used in assessment of IPE.
2. Describe the educational strategies utilized.
3. Describe which aspects of IPC that are being assessed.
4. Explore the theoretical basis for instruments and assessment practice.

## Methods and design

This protocol has been reported in accordance with the Preferred Reporting Items for Systematic Reviews and Meta-Analysis Protocols (PRISMA-P) statement [17] (see PRISMA-P checklist, provided as Additional file 1). The planned systematic review will be reported in accordance with the PRISMA statement [18]. This review protocol has been pre-registered within the Open Science Framework [https://osf.io/vrfjn].

### Eligibility criteria

Studies will be selected according to the criteria outlined below.

### Types of studies

We will include studies with a quantitative (e.g. experimental studies, observational studies, quasi-experimental studies) or mixed methods design.

### Population

We will include studies that assess undergraduate students from two or more health professions (e.g. medicine, nursing, occupational and physical therapy, pharmacy, psychology) represented in the educational activity.

### Intervention

Studies with educational interventions assessing one or more aspects of IPC (values/ethics for interprofessional practice, roles/responsibilities, interprofessional communication and teams and teamwork). Furthermore, they must have used a self-report instrument (e.g. scales, evaluation form, survey) and evaluated the psychometric properties (e.g. validity, reliability) of such an instrument.

### Outcomes and prioritization

The overall outcome will be the effectiveness of instruments used to assess interprofessional competence. Primary outcomes will be the psychometric properties (e.g. reliability, discriminant and internal validity) of instruments used. Secondary outcomes will include time from intervention to assessment, how items relate to specific performance/competencies (or general abstract constructs) and how scores are used (e.g. to grade students, to improve courses or research purposes).

### Exclusion criteria

The exclusion criteria are:

- Editorial letters, commentaries, review articles and qualitative studies.
- Studies presenting results from both students and practitioners/faculty.
- Studies reporting only on course satisfaction.

### Information sources and search strategy

A literature search will be conducted to identify relevant studies from the following electronic databases: PubMed, Cumulative Index of Nursing and Allied Health literature (CINAHL), Web of Science and ERIC. Peer reviewed articles in the English language, published from January 2010 onwards, will be included in the review. The search strategy is developed using a combination of medical subject headings (MeSH) adapted for each database and abstract/titles using the Boolean operators (OR/AND). Search strings and synonyms will be adapted for each of the three databases using a combination of the following aspects: interprofessional learning, health professions, psychometric properties, assessment of learning and assessment tools. Our search strategy was developed by the research team in collaboration with an experienced information specialist (see Additional file 2: Table S1). To maximize retrieval, reference lists of all included studies will be hand-searched to identify relevant articles missed in the electronic search.

### Data selection and screening process

After completed database searches, results will be uploaded to Covidence™, an online systematic review program to facilitate efficient collaborative study screening and selection [19]. Titles and abstracts will be screened for inclusion using inclusion and exclusion criteria (see below) independently by two researchers (RA, SE). Full-text articles will then be examined in detail and further screened for eligibility. In case of disagreement, a third reviewer (CT) will be used as an arbiter and consensus reached through discussion. We will list excluded studies and reasons for exclusions.

### Data extraction

A data extraction form will be developed and populated with data extracted from each study. Analyses will be conducted, and data presented, in accordance with the review questions. Data extraction will be managed using Covidence™. The extraction form will be piloted, modified and refined based on a sample of studies, first independently by two reviewers (RA, SE) then via consensus.

Data extraction will be conducted independently by two reviewers (RA, SE) in relation to relevance for the research questions. In case of disagreement about data extraction choices, consensus will be reached by involving a third reviewer (CT). Data will be extracted about author/year/country of origin/study design/measurement properties (reliability, validity)/sampling; study participants; intervention activities; underpinning theories; outcome of interventions; and approaches to data analyses.

### Evaluation of study quality

Identified studies’ quality will be assessed using the Medical Education Research Study Quality Instrument (MERSQI) guidelines [20] alongside the Newcastle-Ottawa Scale-Education (NOS-E) [21] and Best Evidence in Medical education (BEME) guidelines [22]. These guidelines, developed to appraise methodological quality in medical education research, will be adapted as necessary to suit our review aims, objectives and retrieved studies. An adaption of the Consensus-based Standards for the selection of health Measurement Instruments (COSMIN) checklist [23] will be used to evaluate measurement properties.

### Data synthesis

The extracted data will be systematically recorded and analyzed using descriptive statistics and narrative synthesis. General information and instrument details will be summarized using tables. Preliminary searches of the literature suggest that instruments, populations, designs and outcomes are likely to be heterogeneous. Accordingly, we do not anticipate statistical meta-analysis being suitable or warranted. Underpinning theoretical frameworks, methodological quality, measurement properties and impact of study interventions will be described and synthesized narratively. An analysis of possible subgroups, e.g. by context (simulation praxis, clinical practice, theory based), or type of IPEC dimension, will be performed using descriptive statistics and narrative synthesis.

## Discussion

Developing, implementing, improving and sustaining IPC for interprofessional practice are an educational challenge. Most interprofessional activities rely on self-report questionnaires, both for developing meaningful IPE activities and assessing IPC in students. This systematic review will clarify both the benefits and limitations of commonly used instruments and serve as a guide for choosing the most appropriate existing self-report instrument to assess IPC based on their psychometric and other properties. Furthermore, the results will inform educational practice on how to design and conduct IPC assessment using self-report instruments. Potential limitations at the study and review level include the variety of IPL activities, study conditions and outcomes found in the included studies—i.e. intervention and study heterogeneity. This may negate the appropriateness of statistical synthesis. Any protocol amendments will be documented in a protocol amendment and in the final manuscript of the systematic review.

## Supplementary information

**Additional file 1:** PRISMA-P checklist.

**Additional file 2:** Table S2

## Data Availability

Not applicable.

## Abbreviations

IPC: Interprofessional competence
IPE: Interprofessional education
IPEC: Interprofessional Education Collaborative
PRISMA-P: Preferred Reporting Items for Systematic Reviews and Meta-Analysis Protocols
CINAHL: Cumulative Index of Nursing and Allied Health literature
MERSQI: Medical Education Research Study Quality Instrument
NOS-E: Newcastle-Ottawa Scale-Education
BEME: Best Evidence in Medical Education
COSMIN: Consensus-based Standards for the selection of health Measurement instruments
