# Supplementary material for: Assessment of interprofessional competence in undergraduate health professions education: protocol for a systematic review of self-report instruments
Source: Syst Rev. 2020 Jun 12;9:142. doi: 10.1186/s13643-020-01394-7 (PMC7293112; doi:10.1186/s13643-020-01394-7)
Supplement: Supplementary file 2 — Additional file 2: Table S2 [file 13643_2020_1394_MOESM2_ESM.docx]

**Additional file 2.**

**Table 1. Search strategy for Pubmed database**

| **Search** | **Search term** | **Search field** |
| --- | --- | --- |
| 1 | Interprofessional relations | Mesh |
| 2 | Interprofessinal OR multiprofessional OR multi-professional | Title/Abstract |
| 3 | #1 OR #2 |  |
| 4 | Physical therapist | Mesh |
| 4 | Physical therapist OR physical therapy OR physicaltherapy OR physiotherapist OR physiotherapists OR nurs* OR medical OR medicine | Title/Abstract |
| 5 | #3 OR #4 |  |
| 6 | Students | Mesh |
| 7 | Students OR student | Title/Abstract |
| 8 | Students medical OR students nursing | Mesh |
| 9 | #6 OR #7 OR #8 |  |
| 10 | Questionnaire OR rubrics OR form OR scale OR scales OR survey OR valid* OR reliable OR reliability OR psychometric* | Title/Abstract |
| 11 | Surveys and questionnaires OR Psychometrics | Mesh |
| 12 | Assessment OR assessing OR assess OR outcome OR outcomes OR examin* OR evaluate OR measurement OR measure OR measuring | Title/Abstract |
| 13 | #10 OR #11 OR #12 |  |
| 14 | #3 AND #5 AND #9 AND #13 |  |
